# An evaluation of an intervention designed to help inactive adults become more active with a peer mentoring component: a protocol for a cluster randomised feasibility trial of the Move for Life programme

**DOI:** 10.1186/s40814-019-0473-y

**Published:** 2019-07-09

**Authors:** Andrew O’Regan, Liam Glynn, Enrique Garcia Bengoechea, Monica Casey, Amanda Clifford, Alan Donnelly, Andrew W. Murphy, Stephen Gallagher, Paddy Gillespie, John Newell, Mary Harkin, Phelim Macken, John Sweeney, Mo Foley-Walsh, Geraldine Quinn, Kwok Ng, Nollaig O’Sullivan, Gearoid Balfry, Catherine Woods

**Affiliations:** 1 Graduate Entry Medical School, University of Limerick, Limerick, Ireland; 2 Department of Physical Education & Sport Sciences, University of Limerick, Limerick, Ireland; 3 School of Allied Health, University of Limerick, Limerick, Ireland; 4 NUI and HRB Primary Care Clinical Trial Network Ireland, Galway, Ireland; 5 Faculty of Education & Health Sciences, University of Limerick, Limerick, Ireland; 6 Health Economics & Policy Analysis Centre (HEPA), NUI Galway, Galway, Ireland; 7 School of Mathematics, Statistics & Applied Mathematics Clinical Research Facility, NUI Galway, Galway, Ireland; 8 Go for Life Programme, Age and Opportunity, Dublin, Ireland; 9 Limerick Local Sports Partnership, Limerick, Ireland; 10 Clare Local Sports Partnership, Ennis, Ireland; 11 Limerick City and County Council, Limerick, Ireland; 12 Health Service Executive: Health & Wellbeing Division, Naas, Ireland

**Keywords:** Physical activity, Exercise, Peer mentor, Inactive adults, Train the trainer, Cascade model, Sedentary, Scalability

## Abstract

**Background:**

There is overwhelming evidence to support the promotion of physical activity in adults in terms of benefits to well-being, physical and mental health. Physical activity guidelines suggest that adults should accumulate at least 150 min of moderate to vigorous physical activity per week. In Ireland, the majority of adults do not achieve these guidelines, with costs to health and economy. ‘Move for Life’ (MFL) employs behavioural change techniques delivered by an instructor and peer mentor, using a train-the-trainer (cascade) model. This study will conduct a feasibility cluster randomised controlled trial of the MFL intervention for modifying physical activity behaviours in inactive adults aged 45 years and older.

**Methods:**

The trial is set in eight Local Sports Partnership (LSP) hubs that have structured physical activity programmes. The hubs are the units of randomisation (clusters), and individuals are the units of analysis (participants). Eligible participants will contact one of the hubs, with each hub running four physical activity programmes. Each programme requires between 12–15 inactive adults, resulting in 48–60 participants per hub. Allowing for 20% dropout rate, an additional 96 people will be recruited giving a maximum sample of 576. The hub will be randomised: true control, usual programme or MFL intervention. The true control group will be given information about physical activity but will not be included in a programme for the duration of the trial; the intervention will involve the instructor training one (or more) of the participants to be a peer mentor using an educational toolkit; and usual care groups will have physical activity classes delivered as normal. Baseline data will collect physical activity measures and follow-up measurements will be obtained at 3 and 6 months. All participants will be asked to wear a device for measuring activity on the thigh (activPAL) for 7 days before commencing the programme and at 3 and 6 months. The primary objective of the study is to investigate if it is feasible to deliver the intervention and collect data on moderate to vigorous physical activity (MVPA) on all participants, thereby providing valuable information to guide sample size calculation for a future, more definitive trial.

**Trial registration number:**

ISRCTN11235176

**Electronic supplementary material:**

The online version of this article (10.1186/s40814-019-0473-y) contains supplementary material, which is available to authorized users.

## Background

Being physically active has significant benefits for health and well-being with evidence to show that meeting physical activity guidelines (PAGL) promotes physical and mental health [1–3]. There is evidence that attaining moderate to vigorous physical activity (MVPA) levels that may even be below the recommended 150 min per week can significantly reduce mortality rates, and when the time spent in MVPA is increased, the protective benefits increase accordingly [4]. Age is a significant negative determinant of physical activity, which declines by two-thirds between the ages of 20 and 90 [5]. Despite this gradual decline, those who do engage in physical activity as they age have increased odds of maintaining well-being in later life [6]. Being inactive is associated with a range of poor health outcomes and increased all-cause mortality [7]. Furthermore, physical inactivity in older adults reduces mobility and functional independence [8], with negative consequences for social participation and emotional health. Internationally, the social and economic costs of physical inactivity are recognised but national policies in many countries, including Ireland, to increase physical activity levels remain weak [9]. There is strong evidence to show that complex interventions based on behavioural change strategies can both increase [10] and maintain [11] physical activity levels. Interventions such as these have been shown to reduce the incidence of falls in older adults [12]. Likewise, available evidence shows that interventions aiming to increase social support for participation by fostering positive group dynamics can increase physical activity [13]. Peer-delivered interventions could have considerable, positive public health implications in terms of expanding the reach of such efforts [14].

**Fig. 1:**
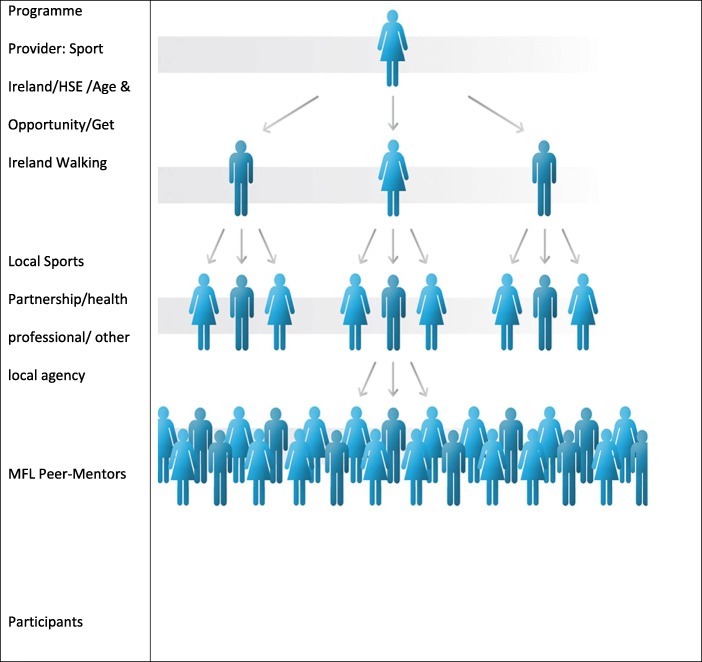
The Move for Life peer mentor model cascading professional impact

Move for Life (MFL) has been developed to broaden the reach of the professional which, in this research programme, involves the Local Sports Partnership (LSP) instructor to the participants via peer mentor support. LSPs are geographically structured and state-funded community-based organisations whose specific purpose is to provide approved and structured exercise classes for the communities they serve, using professional instructors. Move for Life will train the professional instructors to facilitate behaviour change by teaching individual behaviour change skills, implementing social support strategies and creating favourable group dynamics. Peer mentors will be trained in ways to keep the group together for the purpose of continuing their physical activity after the standard LSP programme finishes (Fig. 1). If effective, it will maximise the impact of PA (physical activity) programmes, run by Local Sports Partnerships (LSPs), increasing the likelihood of real change, sustainability and scalability. Cascading models (also commonly called train-the-trainer models) have the potential to be time and cost-effective and are widely used in medicine [15] and prevention [16, 17]; however, this method requires rigorous examination [18] in community-based physical activity interventions.

Move for Life aims to augment and complement existing physical activity programmes in counties Clare (with a population of 117,000) and Limerick (with a population of almost 200,000) in the Mid-West region of Ireland initially. The University of Limerick, where most of the academic team is based, has research links with the Local Sports Partnerships of counties Limerick and Clare. These counties cover one whole Health Service Executive Community Health Organisations (HSE CHO) area. These counties, both with distinctive urban dwelling and rural populations, were selected both for convenience to the academic location and for their potential to replicate the national demographic.

A well-designed, rigorously conducted randomised controlled cluster trial (RCT) is required to provide evidence of potential for a comprehensive MFL intervention. Prior to such a definitive trial, a feasibility study is required. MFL will provide this using a feasibility cluster RCT design; it will advise the selection of RCT primary and secondary outcomes and develop our understanding of the impact on hard to reach inactive adults from the professional-peer-participant cascading model of intervention. The purpose of MFL is to increase the daily time spent in MVPA among inactive people aged 45 and over.

The study hypothesis is that it is feasible to deliver the MFL intervention and to measure the time spent in daily MVPA by participants. Therefore, the primary aim is to investigate the feasibility of the trial and data collection. Specific objectives are to record numbers recruited, retention and attrition rates, safety and acceptability of the intervention and measurements and follow-up testing attendances and participant compliance. The secondary aim is to obtain preliminary information on the economic impact of the trial on participants. Accordingly, secondary objectives are to collect data on health costs and quality of life for participants in each arm of the study for the duration of the trial (Fig. 1).

## Methods

The trial will be reported according to the CONSORT extension for randomised pilot and feasibility trials [19] (see Additional file 1 for the CONSORT checklist). The following phases will be run sequentially: screening, enrolment, allocation, follow-up and assessment.

### Study design

Move for Life is a feasibility cluster randomised control trial (RCT) where the LSP hubs are the units of randomisation (the clusters), and individuals within the hubs are the units of analysis (the participants). The advantage of a cluster RCT is that it overcomes contamination problems that arise if a simple random allocation is used. When testing a complex intervention, randomisation of participants risks contamination if individuals from both arms attend the one group. This study will enrol a total of eight hubs and randomise physical activity groups to each of three arms. This framework is in line with the recommendations of Eldridge et al. [20] and the CONSORT [19] guidance on the minimum number of clusters required to obtain accurate estimates of rates and proportions in pilot and definitive cluster RCTs, respectively. The first arm is the Move for Life intervention (three hubs), the second is the usual programme (three hubs) and the third is the true control (two hubs).

### Study overview

**Fig. 2:**
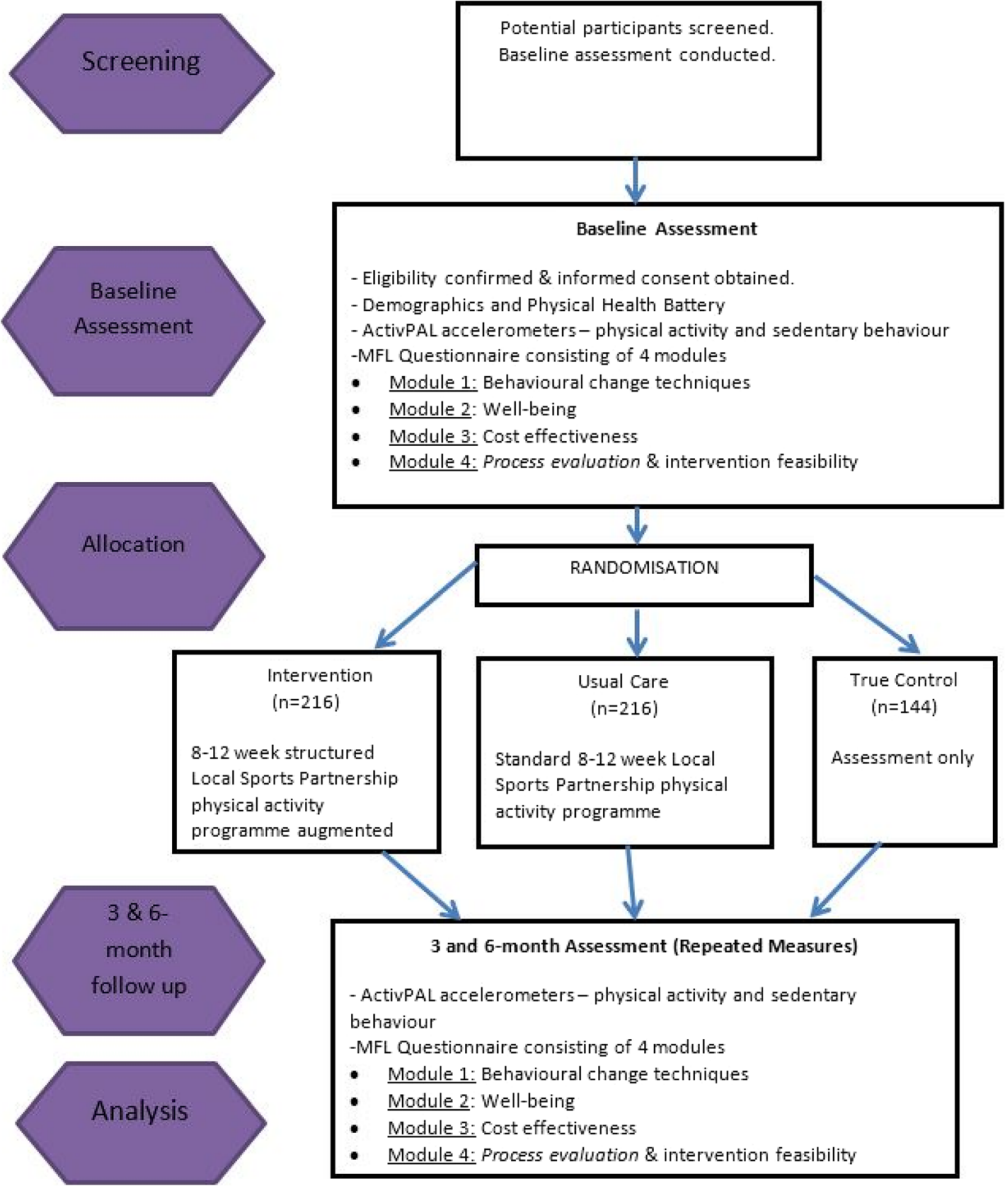
CONSORT Flow diagram of study design

The trial is set in eight Local Sports Partnership (LSP) hubs that have ongoing structured physical activity programmes. LSPs are state-funded community-based organisations whose specific purpose is to provide approved and structured physical activity classes for the communities they serve, using professional instructors. Eligible participants will contact one of eight regional hubs, with each hub running four physical activity programmes. These include Men on the Move, Women on Wheels, Go for Life and Get Ireland Walking. Each hub will be randomised to one of the following: true control, usual programme or MFL intervention. The intervention will augment existing programmes by training professional instructors to implement individual and group-based behaviour change strategies, assisted by peer mentors that will help keep the group together after the lifetime of the programme. The true control group will be given information about physical activity but will not be included in a programme for the duration of the study; usual programme groups will have physical activity classes delivered as normal. Baseline data will be collected on physical activity measures and follow-up measurements will be obtained at 3 and 6 months. Participants in all groups will be asked to wear a lightweight device for measuring activity on the thigh (an activPAL) for 1 week before commencing the programme and again at 3 and 6 months. An Adverse Events Reporting System will be in place, reporting back to the Move for Life advisory committee, who will make decisions about trial safety and continuance. Ethical approval for the study was granted by the University of Limerick, Faculty of Education and Health Sciences Research Ethics Committee (reference number—EHS_2018_02_15, approved on April 9, 2018) (Fig. 2).

### Study setting

The study will take place in the Health Service Executive Mid-West region of Ireland in pre-existing community sport and physical activity hubs that were developed as part of Ireland’s national physical activity plan and whose purpose is to increase engagement in physical activity generally and particularly amongst disadvantaged, marginalised and hard to reach groups. A total of eight hubs across Clare (*n* = 4) and Limerick (*n* = 4) will be enrolled. Data collection will be entirely completed in a community setting, using the community or parish halls in which the exercise programmes are conducted. The study is being conducted by a multidisciplinary team from the University of Limerick, Faculty of Education and Health Sciences, and the Local Sports Partnerships, in collaboration with NUI Galway, Health Services Executive Health and Wellbeing, Healthy Ireland, Age and Opportunity and Clare, Limerick city and county councils.

### Sample size justification

As this is a feasibility study, a formal sample size calculation has not been used but the sample size was estimated based on the primary outcome of interest, which is the feasibility of measuring MVPA. The minimum numbers required to sustain structured physical activity programmes and necessary group dynamics. Each programme involves an instructor working with a group and, for feasible group dynamics, requires between 12–15 inactive adults. If each hub involves four physical activity programmes, it will give a sample of 48–60 participants per hub—a total of 480 participants. Allowing for a 20% dropout rate, an additional 96 people will be recruited giving a maximum sample of 576.

### Participants: eligibility and recruitment

Recruitment will start 8 weeks before the trial commences. The study aims to recruit inactive, harder to reach groups including ethnic minorities, people less inclined to group participation and those that are socially excluded. ‘Physically active’ is defined by the national Physical Activity Guidelines (PAGL) as 150 min of physical activity of at least moderate intensity per week [21, 22]. All potential participants will be asked to complete a short two-item self-report physical activity screening questionnaire [23]. Those who are aged over 45 and who report less than 150 min of MVPA will be invited to participate in the physical activity programme. The cut-off range of 45 years was chosen as it became apparent that few adults aged between 50 and 60 were registering an interest in participation when the study was advertised for ‘older adults’. The trial will be restricted to adults aged 50 +. Also, potential participants must be able to undertake exercise independently.

The study collaborators, Limerick and Clare Local Sports Partnerships, Healthy Limerick, Age Friendly Limerick/Clare and the Health Services Executive, Age and Opportunity, will assist with recruitment. These collaborators have committed to recruiting participants through community networks and have an established track record in recruiting inactive adults aged fifty and over. Further recruitment through the media, local parish newsletters, sports and community organisations including sports clubs like the Gaelic Athletic Association, active retirement group and others will be involved in recruitment. Non-traditional recruitment methods including liaison with local charities and advertisements with mental health services will be used to recruit marginalised groups. Primary health care professionals including GPs, physiotherapists, pharmacists and nutritionists will be incorporated into the recruitment strategy. Evidence suggests that advice from these groups promotes engagement in physical activity programmes [24–27]. An official MFL launch, addressed by the president of the university and a government minister, will take place 2 weeks prior to trial commencement and will receive local and national media coverage. Social media will also be used and MFL Twitter and Facebook accounts will be used for recruitment.

The inclusion and exclusion criteria for the trial are listed below:

Inclusion criteria:

- Inactive adults, i.e. adults who self-report that they do not meet the PAGL of 150 min of exercise moderate to vigorous physical activity per week
- Community dwelling
- Age 50 years and over
- Able to exercise independently

Exclusion criteria:

- Aged under 50 years
- Active adults, as defined above
- Unable to exercise independently

### Randomisation process

The hubs will be the unit of randomisation and will be randomised to each arm. Each hub (cluster) will be geographically separated to reduce contamination effect. Clusters will be stratified as rural or urban. Hubs will be randomised to one of the following: true control (two hubs), usual programme (three hubs) or MFL intervention (three hubs). Participants in the true control group will be given information about physical activity but will be placed on a waiting list for access to the MFL physical activity programmes for the duration of the trial. They will be offered the programmes after the trial. The participants allocated to the MFL intervention group and the usual physical activity programmes will not be aware of their status. Randomisation will only occur and will only be revealed to participants and their instructors after all the baseline data have been collected.

### Description of comparator intervention

The physical activity programmes in the comparator intervention will be run by the LSPs at existing exercise hubs in community settings. The programmes were developed as part of Ireland’s national physical activity plan, ‘Get Ireland Active’ [28]. These programmes run nationally and their purpose is to encourage and support physical activity by facilitating access to a wide range of physical activity opportunities in local communities. For Move for Life, four existing LSP programmes were identified as suitable for inactive adults aged 45 + years. Two programmes are gender segregated: ‘Men on the Move’ and ‘Women on Wheels’, both are 12-week structured exercise classes involving general sport, physical activities and cycling. ‘Get Ireland Walking’ is an 8-week walking initiative for inactive men and women; ‘Go for Life’ is an 8-week structured exercise class that typically recruits older adults aged 65 years plus. For the purposes of the comparator intervention in this trial, these programmes will run in the usual way so that participants are exposed to a community-based structured physical activity programme. Participants in the intervention arm of the trial will have these programmes augmented by the Move for Life intervention.

### Development of the intervention

Three hubs will be allocated to the MFL intervention arm. Documents relevant to the four LSP programmes to be augmented for MFL were examined. In 2018, a qualitative study, involving stakeholders in current community exercise programmes, was conducted to investigate factors that affect recruitment, retention and scalability of these programmes.

Using the sources outlined, the MFL intervention was designed on a foundation of theoretical, empirical and practical information. The intervention mapping approach was used to identify theory-based determinants and matching them with appropriate methods for change [29]. A toolkit in the form of a training manual has been developed to train MFL professional instructors in key behaviour change techniques, involving social support and group dynamic strategies along with behavioural skills, aimed to facilitate change and maintenance of physical activity over time. The MFL toolkit contains strategies and outcomes identified in the intervention mapping process. The process will be described in more detail in a separate paper. Social cognitive theory (SCT) [30] was the primary conceptual framework of the intervention because of its emphasis on self-efficacy and social support. Behavioural skills strategies to address outcome expectancy and self-efficacy determinants of behaviour were derived from previous SCT-based intervention work [31, 32] The Theory of Planned Behaviour [33], a second conceptual framework, guided the intervention design in terms of influencing participants’ attitudes and beliefs around physical activity. Self-determination theory (SDT) [34] was a third conceptual framework, using its focus on basic psychological needs for autonomy and relatedness to others to having a positive impact on desired behavioural outcomes [35]. A fourth conceptual framework, the model of group cohesion in exercise and sport [36], was employed, with particular emphasis on task cohesion (i.e. how well participants work together toward a common goal) and social cohesion (i.e. how much participants enjoy working with each other toward the goal) to develop strategies for the group to achieve its outcomes.

It was initially envisaged that the Local Sports Partnerships (LSP) tutors would identify a suitable non-professional volunteer from existing programmes to act up as a peer mentor for this study. The desirable criteria were strong motivation and interpersonal skills and experience with group dynamics. However, because of challenges recruiting in this way and concerns about upsetting the group with an ‘outsider’ as a peer mentor, it was decided to recruit the peer mentor from the groups during the trial. Part of the role of the LSP tutor facilitating the programme would be to identify participants suitable for the peer mentor role*.*

A team of educational experts with experience in physical activity and behavioural change will provide the peer mentor training through a series of interactive workshops. The peer mentors will be identified by the LSP sports development officer in the early stages of the Move for Life trial and will receive training during the course of the programme. They will be trained in motivational change techniques so that they will be able to develop rapport with their peers and to support and encourage them in their journey to become more physically active. They will be trained on group dynamics, how to access local assets and facilities, to access funding by applying for grants and to promote their group by social and local media so as to secure the sustainability of the programme. During the trial, they will have the support of the sports development officer and, thereafter, peer mentors will be provided with leadership training courses and linked with local community organisations, including the LSPs.

The MFL peer mentor will receive training from the MFL team, which will provide advice on how to maintain the group post 8/12 weeks formal programme. This training will provide information and organisational support and identify social and environmental supports for physical activity locally. Additionally, during the MFL programme, individuals who show an interest in helping to sustain the programme, post involvement of the professional, will be identified by the MFL instructor and the peer mentor, and these emerging assistants will be provided with support to contribute to the sustainability of the programme. At the end of the MFL programme, the assistants will receive training to become a new MFL peer mentor. An ‘augmented’ physical activity program has been conceptualised with a clearly defined behaviour change focus and related activities for each week. It can be adapted for 8, 10 or 12 weeks depending on the duration of the particular MFL programme strand. It will not be possible to blind the professional instructors as they will have to learn the techniques developed for the MFL intervention. Final details of the intervention are currently being completed by a multidisciplinary team.

## Outcomes

The primary outcome of the study is to investigate the feasibility of measuring MVPA. The acceptability and safety of the intervention and measurement methods will be investigated. The data will provide reliable estimates for sample size calculation for a future definitive trial. Secondary outcomes relate to data on recruitment, allocation, retention, attrition and attendance. Data will be collected on safety and adverse events will be recorded by the LSP tutor. Participant recruitment outcomes include demographic profile and the success of each of the recruitment strategies. Participant retention outcomes include the number, profile and reasons of participants that drop out. Data will be collected on the numbers who complete each part of the physical activity measures and questionnaires and who wear and return the activPAL devices. Questionnaires will enable the collection of data on healthcare resource use, out-of-pocket expenses and health-related quality of life. For each secondary outcome, the point estimate of effect will be reported, and precision will be presented with 95% confidence intervals. SPSS version 25 will be used for the statistical analyses. At specific time-points, immediately post intervention and 3 months post intervention, follow-up testing sessions will take place. The authors define ‘minimum thresholds’ in the context of the post intervention and 3-month follow up testing as the minimum proportion of participants that attend the testing sessions and provide reliable data.

### Progression criteria

The feasibility study will progress to a full study unless there is:

- Failure by more than 40% of participants to provide reliable data for daily determination of time spent in moderate to vigorous exercise
- Failure by more than 40% of participants to maintain engagement with the intervention.
- Failure to identify less than 80% of the required number of peer mentors by the LSP tutors in a timely fashion.

### Data collection

The data collection team will conduct the physical tests, apply the activPAL device and facilitate completion of a questionnaire by all participants. After eligibility has been confirmed and informed consent has been obtained, MFL participants will complete a questionnaire comprising of four modules: behavioural change techniques, well-being; healthcare resource use and out-of-pocket expense and demographics (Additional file 3). They will then undergo a set of physical health assessments, including body mass index (BMI) and waist circumference. Validated tests of functional ability including balance and strength testing will be carried out including grip strength and the TUG test. Other parameters for testing include physical function (6-min walk test), falls risk and dual-task ability (dual-task timed up and go test), stand balance (single leg stand) and lower body strength (single and repeated chair to standing).

Physical activity outcomes (MVPA and daily sedentary time) will be measured using the activPAL, a small (9 g) device worn on the thigh. This device adheres to the right thigh, protected by a waterproof dressing. Participants will be asked to wear it continuously for seven days, and it will record physical activity and body posture (sitting, lying, standing or walking) via tri-axial accelerometer technology. The device has been selected because it records both activity and sedentary time with a high degree of accuracy, and it is well tolerated, producing long continuous wear periods [37, 38]. Data will be processed to identify daily time in bed (detected by a proprietary algorithm), daily standing time, daily time spent sedentary, daily time spent in light physical activity and in moderate to vigorous physical activity. Time spent in light and moderate to vigorous activity will be derived from the triaxial accelerometer data using the cut points validated by Powell et al. [39]. These additional behaviours may impact health in an older population. The testing team who take measurements and process accelerometer data will be blinded as to the trial status of each cluster.

**Fig. 3:**
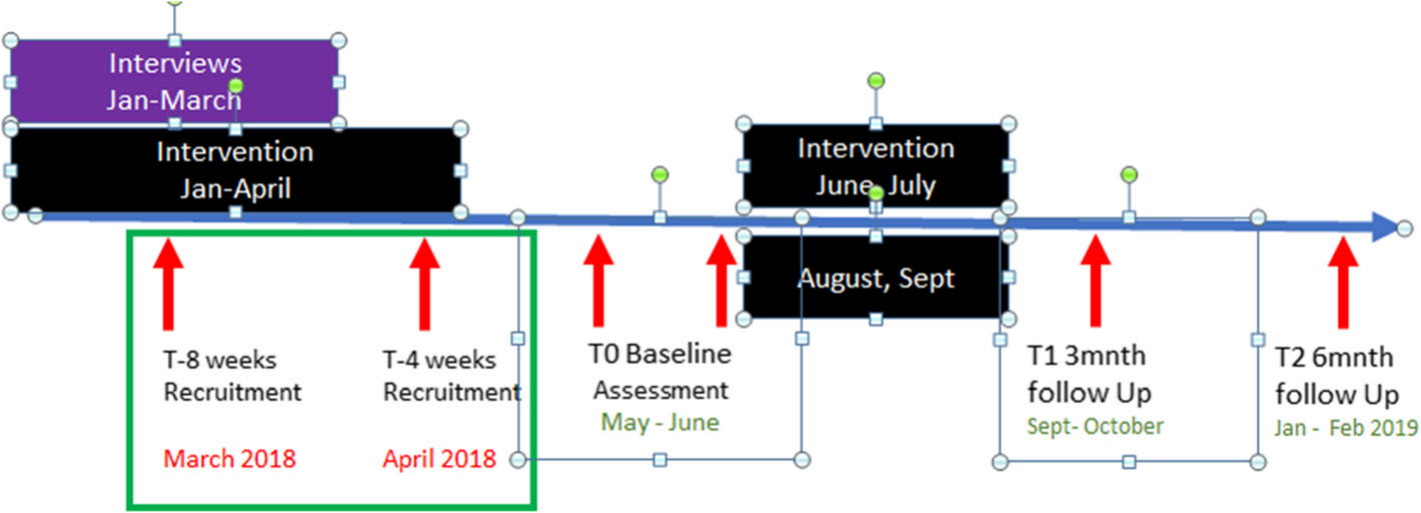
Timeline for the Limerick hubs

Additional file 2 contains the entire testing protocol that will be carried out at baseline, immediately after testing and 3 months post intervention. Information for the process evaluation will be collected on an ongoing basis during the study (Fig. 3).

Interviews of stakeholders involved in previous physical activity programmes were conducted between January and March in order to inform the intervention development. From January to April, the team has developed the intervention. The trial commences in May and 8 weeks before commencing, and recruitment strategies have been developed. Four weeks beforehand, information sessions will take place. The trial will commence with recruitment, enrolment and baseline testing in Limerick hubs in May until July and in Clare from August until October. Follow-up testing at 3 and 6 months will follow for the hubs in Limerick and Clare.

### Analysis plan

As this is a feasibility study, the analysis will focus on confidence interval estimation. Accordingly, any hypothesis testing or regression modelling will be considered entirely exploratory in nature. Demographics and baseline characteristics of all randomized participants will be summarized for each group as well as overall. Continuous variables will be reported as mean ± standard deviation. Categorical variables will be reported as *n* and percentage. The research team analysing the data will be blinded as to the affiliation of each hub. An exploratory analysis of cost-effectiveness, in terms of estimated cost and quality-adjusted life years (QALYs), will be presented.

### Qualitative analysis

After the trial, a subsection of participants will be interviewed to investigate strengths, weaknesses and factors for scalability. This will form part of a mixed methods approach to evaluate the application of MFL and to understand its potential to impact public health on a national level. A process evaluation questionnaire as well as in-depth semi-structured exit interviews with a subsample of intervention participants and stakeholders will be conducted. Recruitment for the interviews will be purposive and sampling will continue until no new themes or categories emerge from the data. Participants will be asked whether they liked the programme and perceived it to be effective and whether the programme language was acceptable and understandable and their perceptions of the professional instructors and assistants evaluated. Interviews will be digitally recorded and transcribed. NVIVO version 11, a qualitative research software package, will be used to assist the analysis of the data with thematic analysis [40] used to analyse the finding.

## Discussion

Participant recruitment is an essential consideration for the MFL feasibility study. Accordingly, several meetings were held to develop the recruitment strategy. The important questions were what strategies to use for recruitment, what days and times would best suit potential participants for recruitment events and how long before the commencement of the trial should recruitment begin. Further challenges envisaged regarding recruitment are that it may be difficult to attract older adults under the age of 60 years who may be working and who may not identify as being ‘older adults’.

It may be difficult to appeal to all these age groups using the same recruitment strategy, terminology and imagery. Furthermore, it may be difficult to convince health care professionals that the interventions are safe and effective for their patients and that may present a recruitment challenge. Consequently, we have involved clinical members of the research team in the recruitment.

There were also questions about numbers of physical activity programmes within each hub and if adequate numbers were not found to run a particular type of programme, what would happen. The team agreed that all hubs would not necessarily have the exact same number of programmes (some may run with three and others with four, depending on demand). There was also the question of participant preference regarding physical activity programmes. The team agreed that at the screening event, participants would be asked to rank the activity programmes in order of preference and they would be advised that they could not be guaranteed their first preference but every effort would be made to accommodate them.

There were several other points of concern for the research team. Ideally, there would have been stratification in the randomisation process in terms of hub profile: social status, age and gender. After discussion with feasibility trial experts, the team decided to stratify along urban-rural lines only. In Limerick, there are two urban and two rural hubs and, in Clare, there are two rural and two urban hubs. The research analysis team will be blinded. It will be necessary to be aware of concealment at meetings and to conceal information regarding the hubs from the analysis team.

The trial will have staggered commencement which is not unusual for a cluster feasibility trial but may have a seasonal effect. It is anticipated that the shorter daylight hours in Ireland from November to February could impact on the outcomes of hours of physical activity and sedentary time especially with regard to follow-up testing which will be in September for some and in mid-winter for later starters. Using freely accessible online data [41], the results will be correlated to the number of daylight hours.

The study will generate important data relating to recruitment and retention of participants; concealment of randomisation; and acceptability and appropriateness of intervention components, data collection and factors for analysis. The data will determine if a larger RCT is possible and will inform the research team of important considerations when planning a trial of larger magnitude.

## Additional files

Additional file 1: CONSORT checklist. (PDF 66 kb)

Additional file 2: Testing Protocol. (PDF 1055 kb)

Additional file 3: Move for Life questionnaire. (PDF 452 kb)

## Data Availability

The datasets generated and/or analysed during the current study are not publically available due to variables in the data which could identify authors and location of hubs. Data generated after the trial has been completed and will be available from the corresponding author on reasonable request.

## Abbreviations

BCW: Behaviour change wheel
BMI: Body mass index
ICC: Intra-cluster coefficient
LSP: Local Sports Partnership
LSPs: Local Sports Partnerships
MFL: Move for Life
MVPA: Moderate to vigorous physical activity
MVPA: Moderate to vigorous physical activity
PA: Physical activity
PAGL: Promotes physical and mental health
RCT: Randomised controlled cluster trial
RCT: Randomised controlled trial
