# Supplementary material for: An evaluation of an intervention designed to help inactive adults become more active with a peer mentoring component: a protocol for a cluster randomised feasibility trial of the Move for Life programme
Source: Pilot Feasibility Stud. 2019 Jul 9;5:88. doi: 10.1186/s40814-019-0473-y (PMC6615191; doi:10.1186/s40814-019-0473-y)
Supplement: Supplementary file 2 — Testing Protocol. (PDF 1055 kb) [file 40814_2019_473_MOESM2_ESM.pdf]

MOVE FOR LIFE

Promoting Physical Activity for Health

Protocols and Procedures

MOVE FOR LIFE

Promoting Physical Activity for Health

For Adults Aged 50+

Move for Life is a research study being carried out by the University of Limerick in association with Limerick and Clare Sports Partnerships, Age and Opportunity, Health Limerick, Limerick City and County Council and NUI Galway. We are aiming to recruit 600 people from Limerick and Clare who are aged 50+ and would like to become more active. Each area will be provided with four fun physical activity programmes that include: **Women on Wheels**, **Get Ireland Walking**, **Men on the Move** and **Go for Life Games.**

All programmes will run for **8-12 weeks**. It is a great opportunity to meet others and to help you live a more active and healthy lifestyle.

Information Evenings	Date & Time		
**Newcastle West Community Centre**	Tuesday	**17th April**	7:00pm
**Croom Civic Centre**	Wednesday	**18th April**	7:00pm
**Claughaun GAA Club**	Tuesday	**24th April**	7:00pm
**Caherdavin Community Centre**	Wednesday	**25th April**	7:00pm

**Free Health Check/Induction**		**Date & Time**		
**Caherdavin Community Centre**	Monday	**14th May**	**21st May**	6:00-10:00pm
**Claughaun GAA Club**	Tuesday	**22nd May**	**29th May**	6:00-10:00pm
**Croom Civic Centre**	Wednesday	**23th May**	**30th May**	6:00-10:00pm
**Newcastle West Community Centre**	Thursday	**24th May**	**31st May**	6:00-10:00pm

Participant Information

What does it mean to be inactive?

If you are meeting the physical activity guidelines of **30 mins** of moderate to vigorous physical activity **5 days a week** or more then they will be deemed to active for the study.

Age Requirements: Even though it is being promoted as being for inactive adults aged 50+ we are happy for participants from 47 years plus to register for the programme.

Times of Programmes: In relation to programme times and dates. We will run Women on Wheels/Bike for Life, Men on the Move and Get Ireland Walking as evening programmes and Go For Life Games as a morning/afternoon programme. This is important to outline to people as time of day may determine which programme you can commit to attend.

Programmes: Each programme will only run if there are enough participants. Therefore not all hubs may have four programmes running at one time.

Important Dates: The most important dates in relation to this study are the **health check** evenings. We are looking to get **80-100** participants in each hub. There are two health check evenings in each hub. Participants will only be required to attend one.

Health Check: Will consist, of **six** stations, 1: questionnaire and screening, 2.height, weight, waist circumference & grip strength, 3 & 4. Balance tests, 5. 6 minute walk and 6. Activity monitor station.

Hub Incentives: Each person who participates in all **three** health checks will earn €30 towards their hub. Each hub can make up to €2,500 for their hub. The Sports Partnership will manage this money to help sustain activities in the hub.

Registration: Registration occurs online at moveforlife.ie or limericksports.ie. However this is not compulsory a participant may turn up on the screening night without being registered. We would encourage as many people as possible to register so that we could manage numbers effectively.

Promoting to Groups: As this is a research study and we require 80-100 people in each hub we would ask you to use any and all networks available to you to promote Move for Life to help us reach these targets.

*Programmes will run the weeks immediately following the final health check. Exact times are yet to be determine

Centre Protocol:

When participants enter centre they will have their name taken and will be assigned an ID code and name badge, given their data recording sheets and questionnaires that will assess. Then they will be directed to the meet and greet station.Testing will be set up with a circuit styled data collection method. The testing time will be between 18.00-22.00 hours for maximum of 35 participants to complete.

Procedure to be completed in order to include:

1) **Station 1:** Meet and Greet **(Catherine and Monica)**
2) **Station 2:** Height, weight, WC, Grip Strength **(2 researchers)**
3) **Station 3:** TUG, TUGC, SLS **(2 researchers)**
4) **Station 4:** Single chair stand & Repeated chair stand **(1 researcher)**
5) **Station 5:** Six minute walk test **(2 researchers)**
6) **Station 6:** ActivPAL station **(2 researchers)**
7) **Station 7:** Checklist & Appt slip

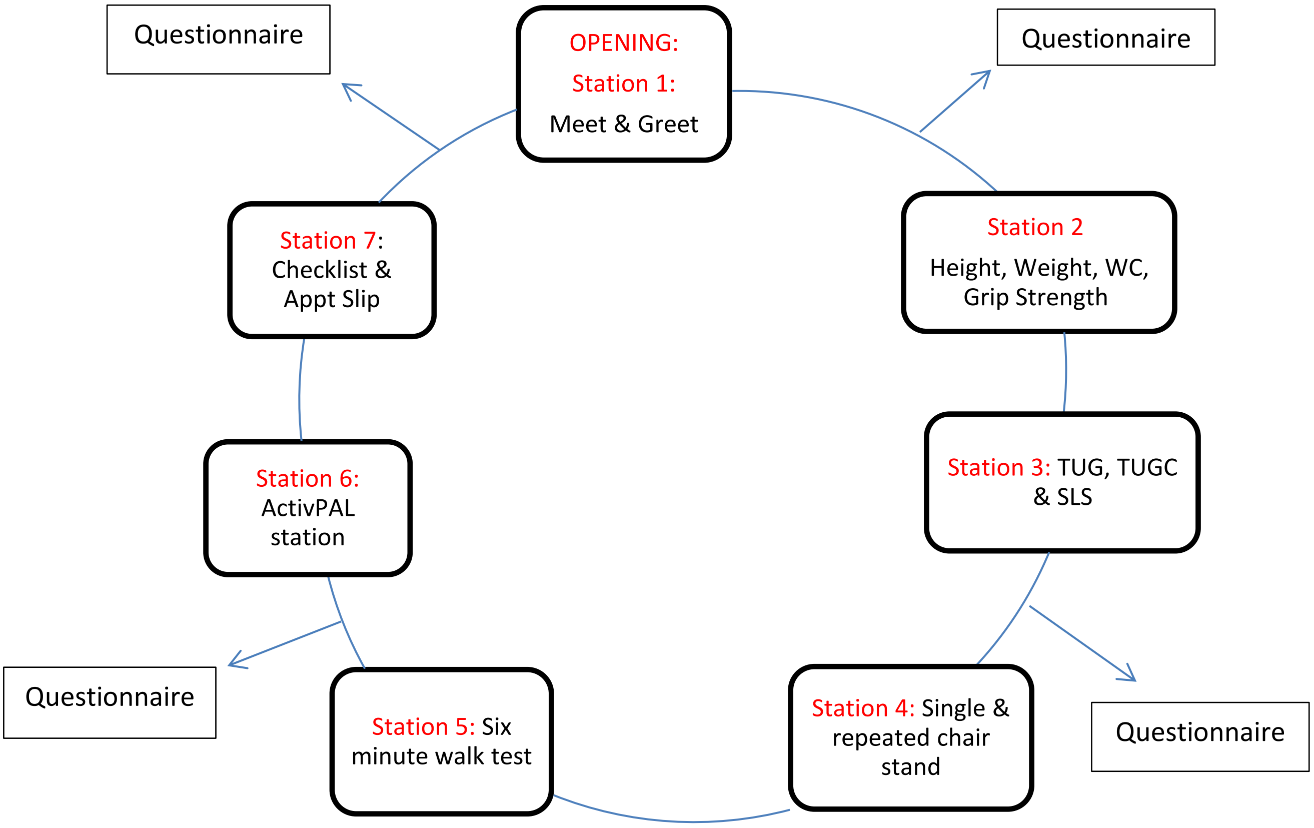

Figure 1. Layout of testing session.

Move For Life Study

Data Collection Checklist

Equipment:

- Weighing scales x2
- Height measure x2
- Grip-strength dynamometer x 3
- Measuring tapes for WC x 4
- Blue screens x 4
- 8 x desks, 14 chairs for testing stations + timed up and go chair x3
- Cones x 20
- measuring tape x 4
- ActivPAL bags
- Stop watches x 5
- Pens x 50
- Labels with name badges
- First aid kit and Emergency phone numbers
- MFL Pop up poster and posterboard (Gearoid has these)

Paperwork:

- Data Recording sheet for all participants
- Questionnaires x 2
- Consent Forms
- Appointment slips & Results CARD
- Limerick LSP programme booklets x 50 for ineligible active people (ask Phelim)
- Description (brief) of 4 programmes x 50 (if there is such a sheet)
- Control group info sheet

Protocol for Physical Measures: Multipurpose Lab

STATION 1: Meet and Greet Station

Equipment:

- Table,
- Chairs x 10
- Consent forms x 60
- Recording Sheets x 60
- Pens
- Questionnaire

Set-up:

- Screening Sheet & Consent. Staple consent to the data recording sheet
- Questionnaire
- If not eligible, information for those participants.

Instructions to participant:

- Ask them to fill out the personal details part of the form: Name, Address, DOB, Contact number

- Ask them to tick which activity they prefer to sign up to; Men on the Move, Women on Wheels, Go for Life.

- Also ask to fill out list of medications here, and identify if it needs to be followed up.

- Then direct them through the stations where free.

STATION TWO: Height Protocol

Equipment:

- Standard collapsible portable stadiometer

Set-up:

- Construct stadiometer following instructions, blue base flat, slot white slats in order.
- Place it on a level, flat, hard surface with the stabilizing bar against a vertical surface such as a wall or door.

Instructions to participant:

- Remove shoes
- If the hairstyle affects their height, ask them to adjust it for the test.
- Ask participants to stand with heels and toes together on the base plate.
- Place arms loosely by their side.
- Make sure that heels, glutes, shoulder blades and head are in line with the vertical measuring rods
- Ask participants to look straight ahead, take a deep breath, and stand as straight as possible without their heels lifting off the ground.
- Allow them to exhale, lower headboard to the highest point of the head and ensure hair is compressed
- Take this recording twice.

Note: These can be difficult instruction for students to follow – make sure the head is not tilted or the shoulders raised, breath normally. Check posture before measuring.

For participants that cannot place heels, glutes, shoulder blades and head against the vertical rod, ensure that at least the glutes and heels or glutes and head are touching the board.

Record in centimetres to nearest 0.1cm

STATION 2: Weight Protocol

Equipment:

- ❖ Standard portable calibrated scales (mechanical)

Set-up:

- ❖ Ensure scales are pre-calibrated with a known weight
- ❖ Place scales on a hard, level surface

Instructions to participant:

- ❖ Wear only light garments
- ❖ Remove items such as keys and money from pockets
- ❖ Remove shoes.
- ❖ Stand on the scales, with both feet equally distributed on the weighing platform, heels towards the back edge, and their arms loosely by their side.
- ❖ Remain as still as possible with their head facing forward.
- ❖ Step down from the scale.
- ❖ Take this recording twice.

Record in kilograms to nearest ½ kilogram

Height and weight will be used to calculate Body Mass Index (BMI)

BMI = Weight (kg) / Height (m^2)

BMI measurements help classify the weight of an individual as underweight, normal, overweight or obese.

References:

- American College of Sports Medicine. (2013). ACSM's guidelines for exercise testing and prescription. Lippincott Williams & Wilkins.
- Canadian Society for Exercise Physiology (1996), Ch1. D Docherty.
- Measurement in Paediatric Exercise Science. (pp18-55) Canada. Human Kinetics

STATION 2: Waist Circumference

Equipment:

- Standard anatomical measuring tape/ blue screen
- Data collection sheets

Set-up:

- The measurements will be taken in an enclosed place so the participant feels comfortable.

Instructions to participant:

- Stand comfortably feet hip width apart, facing tester.
- Pull up and tuck their jumpers or t-shirts so that you can see the naval/belly button.
- Hands by side & Breathe normally.
- They should not contract abdominal muscles.

Procedure:

- Pull a length of the measuring tape, holding both ends in right/ left hand, and bring it around the participant.
- Stretch tape out. Unite both ends at the front by inserting catch. Take slack out of tape by pressing button. (Skin should not be compressed, and there should be NO space between skin and tape).
- Once participant reaches the end of normal expiration, measure the narrowest point of the abdomen, ensuring the tape is level. Make sure you are directly in front of them.
- If no one point is evident, measure half way between the lowest rib and the iliac crest landmark or an inch above the belly button.

Note: If measurements are within 1cm of each other take the average. If it exceeds 1cm, repeat.

Take both recordings twice in cm to the nearest 0.1cm

STATION 2: Grip- Strength Protocol

Equipment:

- ❖ Grip-strength Dynamometer (automated)

Set-up:

- ❖ Ensure dynamometer is calibrated and zeroed before each trial
- ❖ Ensure shoes are removed, level surface
- ❖ Patients with swelling, inflammation, severe pain, or injury should be excluded

Instructions to participant:

- ❖ Give verbal and practical demonstration of the test.
- ❖ Hold dynamometer in **dominant and non-dominant hand**, with hand by side.
- ❖ Adjust handle of dynamometer if required – base should rest on first metatarsal and handle should rest on middle four fingers.
- ❖ Ask participant to keep their upper arm against their trunk, and to avoid any bend in the wrist.
- ❖ When ready, instruct the participant to squeeze the dynamometer with maximal isometric force, maintain squeeze for approximately 5 seconds.
- ❖ No other body movement, if there is flexion or extension on the wrist, repeat the trial
- ❖ Encourage maximal effort.
- ❖ Take 2 measurements on each side.

STATION 3: Timed Up and Go Test (TUG)

Equipment: Arm chair, tape measure, tape, stop watch.

Set Up:

- Begin the test with the subject sitting with their back against the chair, hips all of the way to the back of the seat and their arms resting
- The chair should be stable and positioned such that it will not move when the subject moves from sit to stand. The subject is allowed to use the arm rests during the sit – stand and stand – sit movements.
- Place a piece of tape or other marker on the floor **3 meters away** from the chair so that it is easily seen by the subject.

Procedure:

- When I say "GO" I'd like you to stand up and walk "**as fast as possible yet safe"** to the line on the floor, turn around, walk back to the chair, and sit down again
- Start timing on the word "GO" and stop timing when the subject is seated again.
- The participant wears his/her regular footwear and uses customary walking aid (e.g. none, stick or frame). No physical assistance is given however keep close to the individual if unsteady for safety. There is no time limit. They may stop and rest (but not sit down) if they need to. If the participant does not perform the test correctly e.g. stops at the turn, does not sit down, or does not walk all the way to the 3mt mark repeat the test if able.
- The subject should be given a practice trial that is not timed before testing to ensure the participant understands the procedure.

Record time __________and if any assistive device was used ________

≥14 seconds associated with risk of falls in community dwelling older people

STATION 3: Timed Up and Go (TUG) Test Cognitive

As above but this time the person is asked to perform the TUG while counting backwards in threes from 30. Alternative name as many as **many different types of animals as possible''**

Equipment: Arm chair, tape measure, tape, stop watch.

Set Up:

- Begin the test with the subject sitting with their back against the chair, hips all of the way to the back of the seat and their arms resting
- The chair should be stable and positioned such that it will not move when the subject moves from sit to stand. The subject is allowed to use the arm rests during the sit – stand and stand – sit movements.
- Place a piece of tape or other marker on the floor 3 meters away from the chair so that it is easily seen by the subject

Procedure:

- *"When I say "GO" I'd like you to stand up and walk as "***as fast as possible yet safe***" to the line on the floor, turn around, walk back to the chair, and sit down again while counting backwards in 3's from 30 out loud."*
- Start timing on the word "GO" and stop timing when the subject is seated again.
- The participant wears his/her regular footwear and uses customary walking aid (e.g. none, stick or frame). No physical assistance is given however **keep close to the individual if unsteady for safety**. There is no time limit. They may stop and rest (but not sit down) if they need to. If the participant does not perform the test correctly e.g. stops at the turn, does not sit down, or does not walk all the way to the 3mt mark repeat the test if able.
- The subject should be given a practice trial that is not timed before testing to ensure the participant understands the procedure.

Record time __________and if any assistive device was used ________

STATION 4: Single Leg Stance Test

This test helps us to assess your standing balance.

Equipment: Chair that can be used for support.

Set Up:

- Place a chair or table beside the participant for balance
- Stand on one leg for 30 seconds
- Make sure you are close enough to guard the participant and they understand that they should put their foot down before they fall.

Procedure:

- I want you to stand on one leg for as long as you can, or until I say stop.
- Tester to demonstrate using chair/table/counter/wall for initial support.
- You may hold your foot anywhere, but do not brace your free leg on the standing leg.
- Place your arm by your sides and try not to move your feet or grab a support unless you need to gain your balance.
- When you are ready, pick up one of your feet from the floor and hold it as long as you can. Hold this position for as long as you can, or until I say stop.
- Start timing when hand leaves the chair/table/wall (if you are not using a support, start when the foot is lifted).
- Stop timing when their free foot touches the ground, their hand contacts the chair/table, their foot moved, or 30 seconds has passed.

Record the time to a tenth of a second Right leg (_______)   Left leg (_______)

Cut point Less than 10 sec indicated balance impairment and less than 5 sec may indicate fall risk

STATION 4: Single and repeated chair stand test

Measures the ability of person to rise from a chair. Chair rise is a complex test requiring lower limb strength, range of motion, balance and is included in several risk assessment scales.

Equipment: Stop watch and standard height chair.

Set Up:

- Have the participant sit erect in a standard height chair with the chair back against the wall.
- You can use a chair with or without arms but the participants should **NOT** use their arms.
- *Check that the client can successful perform a single chair rise before you test repeated chair rise.*

Procedure:

- **I'd like you to fold your arms across your chest and stand up one time.**
    - Record Yes or No, is able or not. *If the participant was NOT able* to get up without using arms do not perform the Repeated Chair Rise.
- For the sit-to-stand test with five repetitions subjects are asked to rise from a standard height (43 cm) chair without armrests, five times, as quickly as possible with their arms folded across their chest.
- I'd like you to fold your arms across your chest and when I say go, I want you to ***stand up and sit down as quickly and as safely as you can*** ten times in row**.**
- Record the time from the command "**go**" until the participant is in the final seated position, and the number of completed chair rises (0-5)

- *Inability to rise from a chair five times in less than 13.6 seconds is associated with increased disability and morbidity (Guralnik 2000). The optimal cutoff time for performing the FTSS test in predicting recurrent fallers was 15 seconds*
- *Meta-analysis results "demonstrated that individuals with times for 5 repetitions of this test exceeding the following can be considered to have worse than average performance" (Bohannon, 2006)*
    - *60-69 yrs =**11.4 sec,*** *70-79 yrs =**12.6 sec,*** *80-89 yrs=**14.8 sec***

STATION 5: 6 Minute Walk Test

The aim of this measure is to test walking endurance. It is the distance a person can comfortably walk in 6 minutes at their usual walking speed. 30 meters provides optimum distance (Ng et al. 2013)

Equipment: Stopwatch, tape measure (30m), lap counter/beanbag, 2 cones, chair.

Set Up

- Mark out a standard distance in a straight line with two cones. A distance of 30 metres is ideal.
- Place a chair at the half way distance and at the cones so that the participant can stop to sit if necessary.

Procedure:

- The object of this test is to walk **as far as possible** during 6 minutes. You will walk back and forth between the cones. Six minutes is a long time to walk that quickly, so you will be exerting yourself. You will probably get out of breath and might become exhausted.

- You are permitted to slow down, to stop, and to rest if necessary. If you do need to rest, take as long as you need, and resume walking as soon as you are able. It is ok to drop out from the test completely if you don't feel well.

- You should pivot briskly around the cones and continue back the other way without hesitation. Now I'm going to show you. Please watch the way I turn without hesitation."

- ***Demonstrate by quickly walking one lap yourself****. Walk and pivot around a cone briskly.* "Are you ready to do that? The stop watch starts the moment the participant moves their foot in order to take a step.

- Remember that the object is for everyone to walk **AS FAR AS POSSIBLE** over 6 minutes, but without jogging or running. So if you are walking with friends, don't match their speed and stick together; instead go as fast as you can safely go, since we are trying to measure your own personal result. **START NOW**.

  - ***1st minute****: "You are doing well. You have 5 minutes to go."*
  - ***4 minutes remaining****: "Keep up the good work. You have 4 minutes to go."*
  - ***3 minutes remaining****: "You are doing well. You are halfway done.*
  - ***2 minutes remaining****: "Keep up the good work. You have only 2 minutes left.*
  - ***1 minute remaining****: "You are doing well. You only have 1 minute to go.*
  - ***15 seconds to go****: "In a moment I'm going to tell you to stop. When I do, just stop right where you are and I will come to you."*
  - **At the end of 6 minutes** the participant is told to stop. The assessor places the temporary marker (beanbag) at the stopping point then helps the participant sit down.

- Measure and record the distance between last cone and bean bag. Record number of rests, including time stopped and duration of stop.

Recording the distance

The distance from the temporary marker to the last cone is measured and the distance tallied up according to the number of laps recorded on the counter.

STATION 5: 6 Minute Walk Test

Absolute Exclusion Criteria for 6MWT

Absolute contraindications for the 6MWT include the following: **unstable angina during the previous month and myocardial infarction during the previous month**. Note that we won't be taking a medical history (other than the PAR-Q); however if you were asked about these by a patient (I had a heart attack 4 weeks ago, is it ok to do the test?" or "I have really bad angina; should I still do this?") then we should tell them not to participate.

Reasons for immediate test termination (for any individual) (any one of the following)

(1) chest pain
(2) intolerable dyspnea, (i.e. extreme breathlessness)
(3) leg cramps
(4) staggering
(5) diaphoresis (i.e. abnormal levels of sweating)
(6) pale or ashen appearance

STATION 7: Final Station

Equipment:

- Appointment slips: Next weeks appointment & LSP Contact Details
- Notepad and pen for recording.

Set-up:

- Chair x2.
- Table facing chair x2

Instructions to participant:

- Thank you for participating.
- You can drop off your activPAL and any spare supplies in your activPAL bag with your name printed on the front and your questionnaire next week and specify the time and date.
- Ask them if they have any questions, feel free to ask them.

Procedure:

- Collect data sheet.
- Ensure you have all measurements taken, have contact details in terms of address and phone number
- Ensure you have programme preference recorded.
- Thank them for their time and patience
- Give them the appointment sheet for the following evening.
- Have a good evening.

ActivPAL Information:

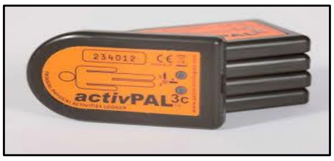

Material needed:

1 x Nitrile sleeve

1 x Waterproof dressing

Instructions:

1. Starting at the rounded top, roll the sleeve down the activPAL (1).
2. Once rolled all the way down (2), cut off the bottom part of the sleeve (3).
3. Fold the sleeve up (4) until it is level with the device (5).

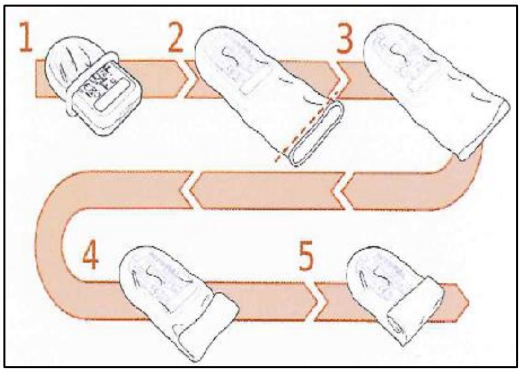

4. Peel back of waterproof dressing (1).
5. Place device (in sleeve) along the centre line, with folded end facing in (2 – 3).
6. Attach device to your mid right thigh (rounded side towards the top) and press in (4).
7. Peel the outer layer of the dressing off and flatten against your skin (5).
8. Ensure that there are no gaps between your skin and the dressing (6).

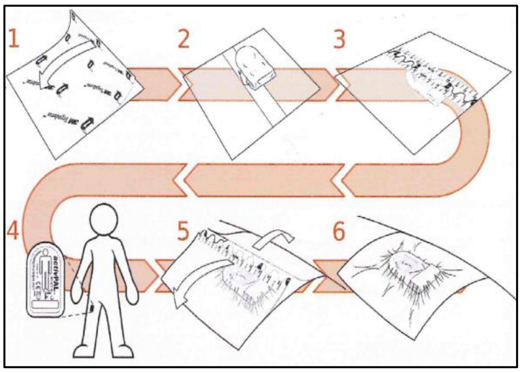

PLEASE record on the attached activPAL diary the time and day that you took the activPAL off and put the activPAL on in your activPAL Diary.

Frequently Asked Questions

Where should I apply the activPAL^{3M}?

It is most comfortable to wear the activPAL on the mid line of the right thigh, about half way down between the hip and the knee.

What if the activPAL^{3M} is not secure?

Ensure that you have the correct side of the bandage sticking to your leg. The sticky side of the bandage will not work well if your leg is wet, or if any lotions (moisturiser or fake tan for example) are on the leg. If the bandage comes loose, you may need to replace it. We have also provided you with a tubigrip bandage that you can wear over the device and a mefix bandage that can also be put over the device when you are exercising. You MUST replace the clear bandage every 3 days or if it begins to get loose. must bring your activPAL and any remaining contents of the bag with you to the collection point in 7 days.

When should I remove the activPAL^{3M}?

The activPAL can be worn comfortably all day and all night and should not impede normal activities. It can be kept on while taking a shower. However, it **MUST** be removed before taking a bath and swimming or before any other activities which may mean the activPAL could come into contact with water for a prolonged period. When taking off the activPAL for any period of time remember to place it on a **flat surface horizontally for the duration that it is off.**

How do I know the activPAL^{3M} is working?

The activPAL is a continuous recorder so will never stop recording. But you can be sure by checking the little light in the front panel. The light will flash green every six seconds. If it is not flashing green please ring **Gráinne** immediately!

What if I lose the activPAL^{3M}?

Don't panic. If you lose or misplace the activPAL, please ring **Gráinne IMMEDIATELY**. She will then try to retrace your steps with you, and will help you look for (and hopefully find) the activPAL. activPAL's are not cheap (each one costs €500), so it is very important that you contact **Grainne** as soon as you have misplaced the device!

What if I'm in trouble or have a question?

At any stage, day or night, if you are having a problem with the device or have a question please give us a ring or text. No matter how small or silly you think the question is, it could turn out to be very important later on.

Gráinne Hayes Mobile Number: 086 0806576

activPAL Diary

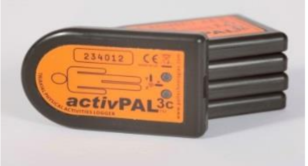

NAME: ____________________

ID NUMBER: ____________________

Date	Day	Time Taken Off	Activity	Time Replaced
04/02/2014	*Wednesday*	*14:58*	*Swimming*	*15.38*

Date	Day	Time Taken Off	Activity	Time Replaced
04/02/2014	*Wednesday*	*14:58*	*Swimming*	*15.38*

Testing Participant Recording Sheet:

Participant Name: _________________________ **Participant ID:** _______________________

Station Two:

A: Height (m): _______ _______

B: Weight (kgs): _______ _______

C: Waist Circum (cm): ________ ________

D: Grip Strength (kgs): **Dominant hand circle**: Right /Left: __________ _________

Non-Dominant hand circle: Right /Left: __________ _________

Station Three:

A: TUG (sec): ______________

B:TUG-Cognitive (Dual Task Ability) (sec): ___________

Station Four:

A: Single Leg Stance (sec): **Dominant Leg circle** (Right/Left)

Right: ____________ Left: _______________

Station Four:

**Single Chair Stand Test**<br>Yes No<br>A. Safe to stand without help ❒ ❒<br>B. Results: Participant stood without using arms ❒<br>→ Go to Repeated Chair Stand Test<br>Participant used arms to stand ❒ → End test; score as 0 points<br>Test not completed ❒ → End test; score as 0 points	C. If participant did not attempt test or failed, circle why:<br><br>Tried but unable: 1<br>Participant could not stand unassisted: 2<br>Not attempted, you felt unsafe: 3<br>Not attempted, participant felt unsafe: 4<br>Participant unable to understand instructions: 5<br>Other (Specify): 6 ____________________<br>Participant refused: 7
**Repeated Chair Stand Test**<br>Yes No<br>A. Safe to stand ten times ❒ ❒<br><br>B. If ten stands done successfully, record time in seconds.<br><br>Time to complete ten stands _____.___ sec	C. If participant did not attempt test or failed, circle why:<br><br>Tried but unable: 1<br>Participant could not stand unassisted: 2<br>Not attempted, you felt unsafe: 3<br>Not attempted, participant felt unsafe: 4<br>Participant unable to understand instructions: 5<br>Other (Specify): 6 ____________________<br>Participant refused: 7

Station Five:

Six minute walk test:

Distance completed in 6 minutes (meters): ________________

Station Six:

ActivPAL: Fitted circle Yes / No

Station Seven: Checklist:

Personal Information		Single leg stance	
Height/Weight/BMI		6 Minute Walk Test	
Grip Strength		ActivPAL Bag	
TUG,		Questionnaire	
TUGC,		Activity Preference	
Single chair stand/ Repeated chair stand		Appointment Slip	

Appointment Cards

MOVE FOR LIFE<br>Promoting Physical Activity for Health<br><br>Name:<br><br>Next Appointment:	MOVE FOR LIFE<br>Promoting Physical Activity for Health<br><br>Name:<br><br>Next Appointment:
MOVE FOR LIFE<br>Promoting Physical Activity for Health<br><br>Name:<br><br>Next Appointment:	MOVE FOR LIFE<br>Promoting Physical Activity for Health<br><br>Name:<br><br>Next Appointment:
MOVE FOR LIFE<br>Promoting Physical Activity for Health<br><br>Name:<br><br>Next Appointment:	MOVE FOR LIFE<br>Promoting Physical Activity for Health<br><br>Name:<br><br>Next Appointment:

MOVE FOR LIFE

Promoting Physical Activity for Health

Name:

Next Appointment:

MOVE FOR LIFE

Promoting Physical Activity for Health

Name:

Next Appointment:

MOVE FOR LIFE

Promoting Physical Activity for Health

Name:

Next Appointment:

MOVE FOR LIFE

Promoting Physical Activity for Health

Name:

Next Appointment:

MOVE FOR LIFE

Promoting Physical Activity for Health

Name:

Next Appointment:

MOVE FOR LIFE

Promoting Physical Activity for Health

Name:

Next Appointment:

NAME and ID Assignment

Number	NAME	ID
Example	Grainne Hayes	H1_999_GH
1		H1_100_
2		H1_101_
3		H1_102_
4		H1_103_
5		H1_104_
6		H1_105_
7		H1_106_
8		H1_107_
9		H1_108_
10		H1_109_
11		H1_110_
12		H1_111_
13		H1_112_
14		H1_113_
15		H1_114_
16		H1_115_
17		H1_116_
18		H1_117_
19		H1_118_
20		H1_119_

Number	NAME	ID
22		H1_121_
23		H1_122_
24		H1_123_
25		H1_124_
26		H1_125_
27		H1_126_
28		H1_127_
29		H1_128_
30		H1_129_
31		H1_130_
32		H1_131_
33		H1_132_
34		H1_133_
35		H1_134_
36		H1_135_
37		H1_136_
38		H1_137_
39		H1_138_
40		H1_139_

Number	NAME	ID
42		H1_141_
43		H1_142_
44		H1_143_
45		H1_144_
46		H1_145_
47		H1_146_
48		H1_147_
49		H1_148_
50		H1_149_
51		H1_150_
52		H1_151_
53		H1_152_
54		H1_153_
55		H1_154_
56		H1_155_
57		H1_156_
58		H1_157_
59		H1_158_
60		H1_159_

61		H1_160_
62		H1_161_
63		H1_162_
64		H1_163_
65		H1_164_
66		H1_165_
67		H1_166_
68		H1_167_
69		H1_168_
70		H1_169_

UNIVERSITY of LIMERICK
OLLSCOIL LUIMNIGH

VOLUNTEER INFORMATION SHEET
Move for Life

What is the study about? The purpose of this study is to assess effect of the Move for Life intervention on exercise levels among people aged 50 and over.

What will I have to do? Involvement in this study will require you to take part in a 6-month trial. As part of this trial, you will be randomly assigned into groups that will either i) receive 'usual care' or ii) take part in the M4L intervention or iii) a wait-list control, where you will receive the Move for Life intervention in 6 months.

How will the trial work? If you participate in the trial, you will be asked:

- To wear a very small and light weight device that sticks onto the right thigh for 7 days. The device has been used before and people generally have no problem with it. It is an accurate way of measuring time spent active and still.
- To complete a range of physical health measures and a questionnaire at baseline, 3 months and finally at 6 months.
- These measures include anthropometric measurements such as your height, weight and waist circumference. Your strength will be measured using a hand grip strength test. You will also complete a sit to stand test where you will sit and rise from a chair in a given time frame. Your balance will be assessed and
- You will be asked to complete a questionnaire that asks about your physical activity, health and wellbeing, it will take about 30 minutes to complete.

What are the benefits? The findings of the study will be used to help build capacity amongst LSP staff and local volunteers within your sport and physical activity HUB, this will hopefully improve services in the future.

What are the risks? You might decide that you do not want to participate in the testing because of other commitments or embarrassment. If this happens, you do not have to answer any question you do not wish to.

What if I do not want to take part? Participation in this study is voluntary and you can choose not to take part.

Who else is taking part? Others from your programme and other programmes around Limerick and Clare will be invited to take part in the study.

What if something goes wrong? The testing procedures in this study are used extensively by our research group, and all researchers are competent with the equipment and safety guidelines for use of equipment have been established. Strict exclusion criteria and safety measures will be implemented at all times. If you show any signs of distress at any stage during the research, the procedure will be terminated immediately. We have strict safety protocols, and trained first aiders are on site should anything untoward occur. In the very unlikely event of a more serious occurrence, we will implement appropriate emergency procedures.

What happens to the information at the end of the study? At the end of the study the information will be used to present results. Nobody's name appears in any of the results; instead, codes will be assigned to each person's data to de-identify it. All data gathered from the research will be stored securely and safely by the principal investigator on a password protected computer, for 7 years. After this time, the data will be destroyed.

What if I have more questions or do not understand something? If you have any questions about the study, you may contact any of the researchers. It is important that you feel that all your questions have been answered.

What happens if I change my mind during the study? At any stage should you feel that you want to stop taking part in the study, you are free to stop and take no further part. There are no consequences for changing your mind about being in the study.

Contact name and number of Principal Investigator.

Prof. Catherine Woods,
PESS Dept. University of Limerick,
Tel (061) 202379
Email: Catherine.Woods@ul.ie

Other investigators
Prof Liam Glynn, Prof Stephen Gallagher, Dr. Amanda Clifford, Prof Alan Donnelly, Dr. Enrique Garcia and Dr Andrew O'Regan.

Thank you for taking the time to read this. I would be grateful if you would consider participating in this study.

If you do not wish to take part in the study, no further action is required.

Yours sincerely,

Catherine Woods

This research study has received Ethics approval from the Education and Health Sciences Research Ethics Committee (**quote approval number). If you have concerns about this study and wish to contact someone independent, you may contact**
Chairman, Education and Health Sciences Research Ethics Committee
EHS Faculty Office
University of Limerick
Tel (061) 234101 Email : ehsresearchethics@ul.ie

Privacy Statement

This Privacy Statement governs the use and storage of your personal data. You can see the University of Limerick's Data Protection Policy at www.ul.ie/dataprotection

The University of Limerick is a 'data controller' of the personal data you as a 'data subject' provide to us. We are collecting personal data from research participants in order to undertake research relating to physical activity behaviour change and rely on your consent as our legal basis for processing personal data.

We collect the following types of personal data from research participants:

- Physical test results (including information on your height, weight, waist and hip circumference, strength and mobility).
- Questionnaire information on your background, your attitudes to physical activity, your health and wellbeing.
- Data on your weekly movement patterns collected using a worn device (activPAL).
- Data on your attendance at classes run by your local sports partnership.

Personal data will be processed in the University of Limerick and by our collaborators University College Galway (Prof. Andrew Murphy). Hosting and storage of your data takes place at the University of Limerick, Ireland. We may choose to administer the questionnaire data on electronic tablets at a later stage in the study, and for this purpose we will use software developed by 'Surveyanyplace' and hosted in a secure datacentre in Dublin via Amazon Webservices. No third party providers have access to your data, unless specifically required by law.

Personal data collected for this research project will be pseudonymised (in a manner where the information cannot be attributed to you without additional separate information) within 6 months after the final data collection and will fully anonymised within 12 months after final data collection. All data collated for the purposes of this research project will be retained in accordance with the University's Records Management & Retention Policy (located at www.ul.ie/recordsmanagement).

Should you believe that any personal data we hold that relates to you is incorrect or incomplete, you can request to see this information, rectify it or have it deleted. Please contact Catherine.Woods@ul.ie or via postal address to: Prof. Catherine Woods, Department of Physical Education and Sports Science, Room P1020, University of Limerick.

In the event that you wish to complain about how we have handled your personal data, please contact Data Protection Officer at dataprotection@ul.ie or in writing to Data Protection Officer, Room A1-073, University of Limerick, Limerick. If you still feel that your personal data has not been handled appropriately according to the law, you can contact the Data Protection Authority at info@dataprotection.ie and file a complaint with them.

Volunteer Informed Consent

University of Limerick Move for Life: Cluster Randomised Pilot Trial

Principal Investigator: Prof Catherine Woods, Physical Education and Sport Sciences, Faculty of Education and Health Science, University of Limerick.

Other Investigators: Prof. Liam Glynn, Prof Stephen Gallagher, Dr. Amanda Clifford, Prof. Alan Donnelly, Dr. Enrique Garcia and Dr. Andrew O'Regan.

Introduction to the study: The purpose of this study is to assess effect of the Move for Life intervention on exercise levels among people aged 50 and over.

During the study: All information gathered will be treated in the strictest of confidence. To ensure this, my name will be removed from all the data and replaced with an ID number. Only named researchers on the project will have access to the ID number files. '*Confidentiality of information provided can only be protected within the limitations of the law. It is possible for date to be subject to subpoena, freedom of information claim or mandated reporting by some professions*'.

I have read and understood the information on this form. I have a copy of the volunteer information sheet.

I will take part in a 6-month trial. As part of this trial, I will be randomly assigned into groups that will either i) receive 'usual care' or ii) take part in the move for life intervention or iii) that will receive the move for life intervention in 6 months.

I consent to complete the physical health assessments, to wear a device that will monitor my activity behaviour and to complete research questionnaires at baseline, 3 months and 6 months regardless of group. I understand that I may also be asked to take part in a group discussion or an interview to give my views on what I thought of the programme.

Participants Signature: ______________________

Name in Block Capitals: _____________________

Date: ___________________________________

MOVE FOR LIFE

Physical Activity Readiness Questionnaire

This PAR-Q has been identified as a sensible first step to take if you are planning to increase the amount of physical activity in your life.

Name: ________________________________ **ID:** _______________________ **Age (yrs):** ______________

Please read the following questions carefully and tick the YES or NO option.

Q1. Has your doctor ever said that you have a heart condition and recommended only medically approved physical activity?

☐ Yes **No** ☐

Q2. Do you have chest pain brought on by physical activity?

☐ Yes **No** ☐

Q3. Have you developed chest pain at rest in the past month?

☐ Yes **No** ☐

Q4. Do you lose consciousness or lose balance as a result of dizziness?

☐ Yes **No** ☐

Q5. Do you have a bone or joint problem that could be aggravated by physical activity?

☐ Yes **No** ☐

Q6. Are you or have you been pregnant in the last six months?

☐ **Yes** **No** ☐

Q7. Is your doctor currently prescribing medication for your blood pressure or heart condition? (e.g. tablets, inhaler)

☐ Yes **No** ☐

Q8. Are you aware, through your own experience or a doctor's advice, of any other reason against you exercising without medical approval?

☐ Yes **No** ☐

If you answer yes to one or more of the above questions, you should talk to your GP before undertaking an exercise programme. I have read, understood and completed this questionnaire.

Signed: ___________________ Date: _______________________

SCREENING

Physical activity is any body movement; it can be done at different effort levels.

VIGOROUS effort makes your heart beat faster and you have to breathe deeper and faster than normal. You will probably sweat.

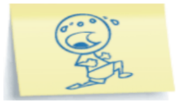

MODERATE effort makes you warmer and your heart rate and breathing rate will be faster than normal. You may also sweat a little, but will be able to carry on a conversation.

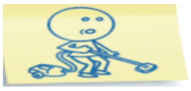

Please think carefully and be as accurate as possible with your answers. Add up all the time you spend in physical activity each day. Only include activities of either moderate or vigorous effort. (Please tick (✓) one box)

Q1. Over a **TYPICAL or USUAL WEEK**, on how many days are you physically active at a **MODERATE** or **VIGOROUS** effort level for a total of **AT LEAST 30 MINUTES** per day?

0 days	1	2	3	4	5	6	7 days

Q2. If 4 days or less, have you done at least 2.5 hours (150 minutes) of moderate effort physical activity over the past week?

**Yes** 1☐	**No** 2☐

Q3. What **HUB** are you going to attend as part of this study? (Please tick (✓) one box)

Caherdavin ☐1	Newcastle West ☐2	Claughan ☐3	Croom ☐4

Q4. Please tick your first preference for the programme that you are interested in attending in this HUB?

Women on Wheels	Go for Life	Get Ireland Walking	Men on the Move
☐	☐	☐	☐

Q5. Please tick your second preference for the programme that you are interested in attending in this HUB?

Women on Wheels	Go for Life	Get Ireland Walking	Men on the Move
☐	☐	☐	☐
