# Supplementary material for: An evaluation of an intervention designed to help inactive adults become more active with a peer mentoring component: a protocol for a cluster randomised feasibility trial of the Move for Life programme
Source: Pilot Feasibility Stud. 2019 Jul 9;5:88. doi: 10.1186/s40814-019-0473-y (PMC6615191; doi:10.1186/s40814-019-0473-y)
Supplement: Supplementary file 3 — Move for Life questionnaire. (PDF 452 kb) [file 40814_2019_473_MOESM3_ESM.pdf]

MOVE FOR LIFE

ID Number: **HUB:** **Date:**

Please take some time to complete the following Move for Life questionnaire

- It will take about 15 minutes to complete. The best approach is to answer each question carefully and focus on each item separately.
- The questionnaire is completely voluntary and while it is important to answer ALL questions, if you should come across a question you don't want to answer, please just move onto the next question.
- Your responses are for **research** purposes only and will be **treated in the strictest confidence,** so try to answer all questions as honestly as you can.

THANK YOU VERY MUCH FOR YOUR TIME AND HELP

The following information will be used to contact you for follow up

Please PRINT all information in CAPITALS

Q1. First Name____ **Q2.** Surname___________________________

Q3. Home postal address ____

Q4. Contact mobile/telephone number ____

Q5. Contact email address____

Q6. How did you hear about Move for Life? (Please tick (✓) all that apply)

Word of Mouth ☐1<br>☐1a Family /Friends<br>☐1b GP<br>☐1c Other	Local Radio ☐2	Parish Bulletin ☐3	Community Newsletter ☐4
Newspaper ☐5	Website ☐6	Information Meeting in local HUB ☐7	Email from Local Sports Partnership ☐8
Other (Please Specify):			

SECTION A: Your Physical Activity (Please tick (✓) one box for each question)

Q1. This question asks you how you feel about doing 30 minutes of moderate effort physical activity on all days or most days of the week for the next 3 months.

	Disagree in a big way	Disagree	Agree	Agree in a big way
1. Doing 30 minutes of physical activity every day for the next 3 months would be **fun**	☐1	☐2	☐3	☐4
2. Doing 30 minutes of physical activity every day would be **enjoyable**	☐1	☐2	☐3	☐4
3. Doing 30 minutes of physical activity every day would be **good for me**	☐1	☐2	☐3	☐4
4. Doing 30 minutes of physical activity everyday would be **important for me**	☐1	☐2	☐3	☐4
5. **My family <u>wants me</u> to** do 30 minutes of physical activity every day	☐1	☐2	☐3	☐4
6. **My friends <u>want me</u> to** do 30 minutes physical activity everyday	☐1	☐2	☐3	☐4
7. **Those whose opinions I value <u>want me</u> to** do 30 minutes of physical activity every day	☐1	☐2	☐3	☐4
8. **My family <u>do</u>** 30 minutes of **physical activity** everyday	☐1	☐2	☐3	☐4
9. **My friends <u>do</u>** 30 minutes of **physical activity** everyday	☐1	☐2	☐3	☐4
10. **Those whose opinions I value <u>do</u>** 30 minutes **physical activity** everyday	☐1	☐2	☐3	☐4
11. **I could be** physically active for 30 minutes every day over the next 3 months if I really wanted to	☐1	☐2	☐3	☐4
12. **I have the time to be** physically active for 30 minutes every day over the next 3 months if I wanted to	☐1	☐2	☐3	☐4
13. **I have a place to be** physically active for 30 minutes everyday over the next 3 months if I wanted to	☐1	☐2	☐3	☐4
14. **I plan to be** physically active for 30 minutes every day over the next 3 months	☐1	☐2	☐3	☐4
15. **I intend to be** physically active for 30 minutes every day over the next 3 months	☐1	☐2	☐3	☐4

Physical activity or exercise includes activities such as walking briskly, jogging, bicycling, swimming or any other activity in which the effort level is at least as intense as these activities.

Q2. How confident you are that you could be physically active in each of the following situations?

	Not at all confident	Slightly confident	Moderately confident	Very confident	Extremely confident
1. When I am tired	☐1	☐2	☐3	☐4	☐5
2. When I am in a bad mood	☐1	☐2	☐3	☐4	☐5
3. When I feel I don't have the time	☐1	☐2	☐3	☐4	☐5
4. When I am on holiday	☐1	☐2	☐3	☐4	☐5
5. When it is raining or snowing	☐1	☐2	☐3	☐4	☐5

Q3. Please **rate how important** each of these statements **is in your decision of whether to be physically active.**

Rate based on **how do you feel <u>right now</u>** not how you have felt in the past or how you would like to feel.	Not at all important	Slightly important	Moderately important	Very important	Extremely Important
**1.** I would be healthier if I exercised regularly	☐1	☐2	☐3	☐4	☐5
**2.** I would feel better about myself if I exercised regularly	☐1	☐2	☐3	☐4	☐5
**3.** Other people would respect me more if I exercised regularly	☐1	☐2	☐3	☐4	☐5
**4.** I would probably be sore and uncomfortable if I exercised regularly	☐1	☐2	☐3	☐4	☐5
**5.** I would feel I was wasting my time if I exercised regularly	☐1	☐2	☐3	☐4	☐5
**6.** My friends and family would get to spend less time with me if I exercised regularly	☐1	☐2	☐3	☐4	☐5

Q4. "The following statements represent different feelings people have when they engage in physical activity. Please answer the following questions by considering **how you typically feel when participating in physical activity** using the scale provided . . ."

	False	Mostly False	More False than true	More True than False	Mostly True	True
1. I am included by others	☐1	☐2	☐3	☐4	☐5	☐6
2. I am part of a group who share my goals	☐1	☐2	☐3	☐4	☐5	☐6
3. I am supported by others in this activity	☐1	☐2	☐3	☐4	☐5	☐6
4. Others want me to be involved with them	☐1	☐2	☐3	☐4	☐5	☐6
5. I have developed a close bond with others	☐1	☐2	☐3	☐4	☐5	☐6
6. I fit in well with others	☐1	☐2	☐3	☐4	☐5	☐6

SECTION B: Your Health Status

Please tick (✓) one box

Q1. In general, would you say your health is…

Excellent □1	Very good □2	Good □3	Fair…□4	Poor…□5

Q2. What about your emotional or mental health? Is it…

Excellent □1	Very good □2	Good □3	Fair…□4	Poor…□5

Q3. Below are some statements about feelings and thoughts. Please tick ✓ the box that best describes your experience of each over the **last 2 weeks**

(Please tick (✓) one box)	None of the time	Rarely	Some of the time	Often	All of the time
1. I've been feeling optimistic about the future	□1	□2	□3	□4	□5
2. I've been feeling useful	□1	□2	□3	□4	□5
3. I've been feeling relaxed	□1	□2	□3	□4	□5
4. I've been dealing with problems well	□1	□2	□3	□4	□5
5. I've been thinking clearly	□1	□2	□3	□4	□5
6. I've been feeling close to other people	□1	□2	□3	□4	□5
7. I have been feeling confident	□1	□2	□3	□4	□5
8. I have been able to make my own mind up about things	□1	□2	□3	□4	□5

Q4. Have you fallen in the past 12 months? Yes □1 No □2

If ***Yes***, in total about how many times have you fallen in the past 12 months? __________number

Q5. Are you afraid of falling? Yes □1 No □2

Q6. Do you have any problems with your walking or balance? Yes □1 No □2

You are half way through, keep going.

Thank You.

Q7. We are interested in your **health-related quality of life**. Please indicate which statements best describe your own health state today.

**MOBILITY**	Please tick (✓) one box
I have no problems in walking about	☐1
I have slight problems in walking about	☐2
I have moderate problems in walking about	☐3
I have severe problems in walking about	☐4
I am unable to walk about	☐5
**SELF-CARE**	Please tick (✓) one box
I have no problems washing or dressing myself	☐1
I have slight problems washing or dressing myself	☐2
I have moderate problems washing or dressing myself	☐3
I have severe problems washing or dressing myself	☐4
I am unable to wash or dress myself	☐5
**USUAL ACTIVITIES (e.g. work, study, housework, family or leisure activities)**	Please tick (✓) one box
I have no problems doing my usual activities	☐1
I have slight problems doing my usual activities	☐2
I have moderate problems doing my usual activities	☐3
I have severe problems doing my usual activities	☐4
I am unable to do my usual activities	☐5
**PAIN/DISCOMFORT**	Please tick (✓) one box
I have no pain or discomfort	☐1
I have slight pain or discomfort	☐2
I have moderate pain or discomfort	☐3
I have severe pain or discomfort	☐4
I have extreme pain or discomfort	☐5
**ANXIETY/DEPRESSION**	Please tick (✓) one box
I am not anxious or depressed	☐1
I am slightly anxious or depressed	☐2
I am moderately anxious or depressed	☐3
I am very anxious or depressed	☐4
I am extremely anxious or depressed	☐5

Q8. To help people say how good or bad a health state is, we

have drawn a scale (rather like a thermometer) on which the best state you can imagine is marked 100 and the worst state you can imagine is marked 0.

We would like you to indicate on this scale how good or bad your own health is today, in your opinion. Please do this by drawing a line from the box below to whichever point on the scale indicates how good or bad your health state is today.

Your health today = ☐

Your own health state today

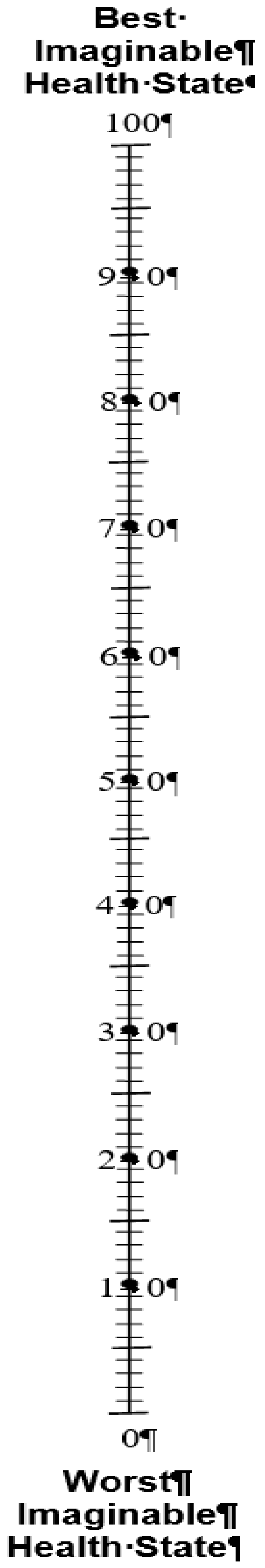

SECTION C: You and your local community *(please tick (✓) one box)*

Q1. Date of Birth: ____/_____/_____ (day/Month/year)

Q2. Gender: Male ☐1 Female ☐2 Other ☐3

Q3 Do you have a **medical card**? Yes ☐1 No ☐2

If ***Yes***, how long have you had a medical card? ____ Months ____ Years

If ***No***, did you lose your medical card in the last year? Yes ☐1 No ☐2 NA ☐3

Q4. Do you have **private health insurance**? Yes ☐1 No ☐2

If ***Yes***, how long have you had health insurance? ____ Months ____ Years

If ***No***, did you give up your insurance in the last year? Yes ☐1 No ☐2 NA ☐3

Q5. How would you describe your present principal status?

At Work ☐1 Unemployed ☐2 Student ☐3

Unable to work due to permanent sickness/disability ☐4 Retired ☐5

Looking after home/family ☐6 Other ☐7 If **Other**, please specify: ____________________

____

Q6. What is your highest level of **education**?

Primary or no formal training	☐1	Lower secondary (e.g. Junior Cert)	☐2
Upper secondary (e.g. Leaving Cert)	☐3	Post-secondary Non-Tertiary (e.g. NCVA level 3)	☐4
Non-Degree (Certificate/Diploma)	☐5	Degree or Higher	☐6

Q7. What is your current **marital status**?

Married/Living with partner ☐1 Widowed ☐2 Separated/Divorced ☐3 Single/Never married☐4

If **Married/Living with partner**, what is your **wife/husband/partner's** present principal status?

At Work ☐1 Unemployed ☐2 Student ☐3

Unable to work due to permanent sickness/disability ☐4 Retired ☐5

Looking after home/family ☐6 Other ☐7 If **Other**, please specify: _________________

____

Q8. Below are statements about living in your neighbourhood.

	Not at all	Just a little	Moderately well	Extremely well
1. In general, how well do you know your neighbours?	☐1	☐2	☐3	☐4

	Like it a lot	Like it a little	Neither like nor dislike	Dislike it a lot	Don't know
2. How much do you like living in your neighbourhood?	☐1	☐2	☐3	☐4	☐5

	Very safe	Fairly safe	Neither safe nor unsafe	Fairly unsafe	Very unsafe
3. How safe do you feel at home during the day	☐1	☐2	☐3	☐4	☐5
4. At home at night?	☐1	☐2	☐3	☐4	☐5
5. "Out and About" during the day?	☐1	☐2	☐3	☐4	☐5
6. "Out and About" at night?	☐1	☐2	☐3	☐4	☐5

	Very easy	Somewhat easy	Neither easy nor difficult	Difficult	Very difficult
7. How easy, or difficult, is it to use public transport near your house? (getting to the bus, train; how often it comes, where you can go to).	☐1	☐2	☐3	☐4	☐5

	Very walkable	Somewhat walkable	Neither walkable nor unwalkable	Not very walkable	Not at all walkable
8. Overall, how would you rate your neighbourhood as a place to walk? (walkable means pedestrian friendly)	☐1	☐2	☐3	☐4	☐5

Q9. The next questions are about how you feel about different aspects of your life. For each one, please say how often you feel that way.

	Often	Some of the time	Hardly ever or never
1. How often do you lack companionship?	☐1	☐2	☐3
2. How often do you feel left out?	☐1	☐2	☐3
3. How often do you feel isolated from others?	☐1	☐2	☐3
4. How often do you feel in tune with the people around you?	☐1	☐2	☐3
5. How often do you feel lonely?	☐1	☐2	☐3

Q10. Below are questions about your participation in your local community. (Please tick (✓) one box)

	Several times a week	Once a week	Several times a month	Once a month	Less than once a month	Never
1. How often do you participate in any groups such as a sports or social group or club, a church connected group, a self-help or charitable body or other community group or a day care centre?	☐1	☐2	☐3	☐4	☐5	☐6
2. How often do you meet socially with friends, relatives or colleagues?	☐1	☐2	☐3	☐4	☐5	☐6

Q11. Please look carefully at the types of organisations in the following questions and tell us how often did you do **unpaid voluntary work** through the following in the last 12 months?

	Every week	Every month	Less often/ occasionally	Not at all
1. Community and social services (e.g. organisations helping the elderly, young people, disabled or other people in need).	☐1	☐2	☐3	☐4
2. Educational, cultural, sports or professional associations (e.g. the GAA)	☐1	☐2	☐3	☐4
3. Social movements (e.g. environmental, human rights) or charities (e.g. fundraising, campaigning)	☐1	☐2	☐3	☐4
4. Other voluntary organisations	☐1	☐2	☐3	☐4

You have now finished the questionnaire.

Thank You. We appreciate your time and help
